# Two Cases of Sudden Death with Autopsical Examinations

**Published:** 1844-11

**Authors:** Austin Flint

**Affiliations:** Prof. of Institutes, &c., in the Rush Medical College; Buffalo, N. Y.


ILLINOIS

MEDICAL & SURGICAL JOURNAL.

VOL. I. NOVEMBER, 1844. NO. 8.

*Two Cases of Sudden Death with Autopsical examinations.* Reported for the Illinois Medical and Surgical Journal, by AUSTIN FLINT, M.D., Prof. of Institutes, &c., in the Rush Medical College.

CASE FIRST.

*Sudden Death from (probable) sudden effusion at base of Cranium.*—By the politeness of Dr. Sands, attending physician to the alms-house of this county, I was invited to be present at an autopsical examination of the body of a woman who had suddenly died under the following circumstances:

She had been committed as a vagrant on the 17th inst., and was in a state of inebriety when brought to the alms-house. On the 18th, she was dressed and about the wards. Did not report herself as being ill; and no particular notice was taken of her condition. She was heard to say that she believed she should die if she could not have some whiskey. It was supposed by the attendants that she was suffering from the after effects of excessive indulgence in ardent spirits; but her general aspect did not indicate any severe ailment, nor did she apply for medical treatment.

At about 2 P. M. on the 18th, while standing in one of the wards, she suddenly threw herself on a bed nigh at hand, and appeared to those in the room to have "*a fit.*" Neither the physician nor resident pupil were in the house. The keeper was immediately summoned, and arrived at the room in three or four minutes after the attack. He stated that she gave two or three respirations after he entered the room, with long intervals between them, and ceased to breathe. He observed no convulsions.

*Examination* 20 *hours after death.*—Body large, well developed, and with considerable *em bon point.* Age about 30. The face and neck, anteriorly as well as posteriorly, presented deep lividity. This partially disappeared after the thorax was opened, and the large vessels divided.

As the objects of the examination were limited to the discovery of the immediate cause of death, it was proposed first to inspect the heart and other organs of the chest.

In opening the pericardium, a sanguinolent fluid was observed to escape. It was estimated that about two ounces were contained within the pericardium. A slit about half an inch in length was observed in the right ventricle, corresponding to the incision through the pericardium. It was undoubtedly made with the scalpel of the operator, although care was taken to guard against this accident. The walls of the right ventricle were morbidly thin but not softened. The slit had the appearance of having been made with a sharp instrument. There was no ulceration or softening about the aperture, nor any indications of endo-carditis. The general dimensions of the heart were not measured, but estimated to be normal; valves unaffected; no coagula within the cavities. The only morbid appearance was the attenuation of the walls of the right ventricle. This ventricle was probably distended, and being in close apposition with the pericardium, received the point of thc scalpel, giving rise to the sanguineous effusion which exuded after dividing the pericardium.

Lungs engorged throughout; excessively at their inferior portions, and considerably at the superior and anterior portions; otherwise no morbid appearances.

The liver was enormously hypertrophied. It extended quite into the left hypochondrium, and was adherent to the diaphragm over a considerable extent of surface, both in the right and left hypochondrium. Its color was light yellow. The hypertrophy of the yellow portion manifestly predominated. It weighed seven pounds and two ounces.

The stomach was larger than usual; not distended; its internal surface not examined. External appearance of intestines healthy.

The object of the autopsy not being attained, the head was next opened.

The dura mater adhered with great firmness, so that in the separation some injury was done to the brain, occasioning a flow of serosity, the amount of which could not be well estimated.

There existed manifestly considerable effusion at the base of the cranium, between the dura mater and arachnoid. It was sanguinolent, but this may have been owing to the rupture of blood vessels in the removal. Bloody fluid flowed freely from the spinal canal. The brain presented moderate congestion. It is to be borne in mind with reference to this, that the chest was *first* opened, and the large vessels divided, which would tend to diminish the quantity of blood within the cranium. Aside from the congestion, appearance of the brain and membranes healthy. Slight effusion into ventricles.

I should have remarked that fluid blood flowed copiously from the vena cava when divided.

*Remarks.*—The most rational explanation of the sudden death in this case would appear to be—Congestion, and sudden effusion at the base of the brain, compressing the medulla oblongata, causing a cessation of respiration and fatal asphyxia. The congestion and effusion were probably induced by excessive inebriety, and perhaps, in some degree promoted by obstruction to the circulation, resulting from the diminished contractile force of the right ventricle in consequence of its attenuated walls.

CASE SECOND.

*Sudden Death, with large Coagula in the Heart, and effusion at base of Cranium.*—By a singular coincidence, I was invited on the same day, to be present at the autopsical examination of a second case, in which death took place as suddenly as in the other instance. The circumstances were briefly as follows:

A boatman, aged about 35, came into the office of Drs. Wilcox and Rogers of this city; was observed to stagger as he entered; seated himself in a chair and uttered the word "Doctor."

It was observed by Drs. Wilcox and Rogers, who were both present, that his eyes had a fixed, staring expression, and that the pupils rapidly dilated. Dr. Wilcox, perceiving that he was about to fall from his chair, seized him by the arm, and allowed him to slide from the chair to the floor, extended him upon his back, and dashed cold-water in his face, supposing it might be syncope. He gasped two or three times afterwards, his limbs were spasmodically extended twice, and he expired, as estimated, about two minutes after entering the office.

It was ascertained, by evidence at the coroners's inquest, held immediately, that he was of intemperate habits, had suffered much from intermittent fever, and for some days past had had diarrhœa. A person who had seen and conversed with him a short time previous, on the same day, stated that his respiration was hurried and labored; that he complained of distress in the stomach, and that his general aspect was exceedingly bad.

*Examination two hours after Death.*—Body considerably emaciated, face very pallid, and features contracted. Presented the appearance of a subject dead after lingering disease.

Head was first examined.

Adhesion of dura mater of ordinary firmness; meningeal veins moderately congested; sero-sanguinolent fluid escaped from within the dura mater at the posterior portion of head. Care was taken to elevate the head while the brain was being removed, and, as estimated, more than two ounces of sero-sanguinolent fluid was found at the base of the skull. Section of brain presented more red points than usual, otherwise no morbid appearances.

*Chest.*—About an ounce of transparent serum in pericardium. Right auricle contained a firm yellow coagulum of lymph, about the size of a small hen's egg. It seemed quite to fill the cavity. A prolongation extended through the auriculo-ventricular orifice. It was firmly interwoven with the musculi pectinati, so as to be with difficulty detached.

A coagulum having the same appearance, but of less size, existed in the right ventricle, firmly intertwined with the chordæ tendineæ. From this coagulum, a prolongation of about half the caliber of the pulmonary artery, extended upward about half an inch beyond the sigmoid valves. The endo-cardial membrane, both of the right auricle and ventricle was remarkably white, and presented no evidence of disease; the left auricle and ventricle nothing abnormal. Dimensions of heart not measured, but estimated to be below the normal size, if any variation existed. Blood flowed copiously from the cavæ when divided, which was fluid when it first escaped, but in a short time formed loose, dark coagula.

Lungs deeply congested, otherwise healthy.

Liver greatly enlarged; of dark red color; deeply congested with fluid blood. Stomach and other viscera not inspected. The objects of this examination were limited to the discovery of the immediate cause of death.

*Remarks.*—What was the immediate cause of death in this case? Was it obstruction arising from the coagula in the heart, or from effusion at the base of the brain? The coagula without doubt existed for a period, greater or less before death. This is shown, first, by the fact that the blood in the large vessels remained fluid, until the vessels were divided and the fluid escaped into the chest. This incident is interesting, as going to illustrate that the property of maintaining the fluidity of the blood, which is inherent in the vessels during life, is not at once lost after death. But, second, the lymph had evidently been subjected to compression of the heart's contraction for some time, as shown by its solidity, the expression of the coloring matter from it, and its being so firmly interwoven with the columns. The space occupied by the coagulum in the auricle especially, must have occasioned considerable obstruction to the circulation. That this, however, had not long existed, is shown by the fact that there was not hypertrophy, but rather an atrophied condition of the heart. The obstruction may have determined the hypertrophy of the liver; it doubtless did its congested state. The congestion and effusion within the cranium, was also, probably, due to the venous obstruction in a great degree; partly, also, to intemperance; and, perhaps, in part, to the state of great debility and anemia resulting from intemperance, intermittent fever, and the diarrhœa, combined. In the latter point of view, it would constitute nearly what has been termed by some authors, "serous apoplexy."

With regard to the question, whether the sudden death is attributable to the morbid condition of the heart or brain, I regard it as open for discussion.

Buffalo, N. Y., Sept. 19th, 1844

